# *hGATA1* Under the Control of a *μLCR/β-Globin* Promoter Rescues the Erythroid but Not the Megakaryocytic Phenotype Induced by the *Gata1* ^*low*^ Mutation in Mice

**DOI:** 10.3389/fgene.2021.720552

**Published:** 2021-10-11

**Authors:** Fabrizio Martelli, Paola Verachi, Maria Zingariello, Maria Mazzarini, Alessandro M. Vannucchi, Annalisa Lonetti, Barbara Bacci, Giuseppe Sarli, Anna Rita Migliaccio

**Affiliations:** ^1^ National Center for Drug Research and Evaluation, Istituto Superiore di Sanità, Rome, Italy; ^2^ Department of Biomedical and Neuromotor Sciences, University of Bologna, Bologna, Italy; ^3^ Unit of Microscopic and Ultrastructural Anatomy, Department of Medicine, University Campus Bio-Medico, Rome, Italy; ^4^ Department of Clinical and Experimental Medicine, Center of Research and Innovation of Myeloproliferative neoplasms (CRIMM), AOU Careggi, University of Florence, Florence, Italy; ^5^ Department of Veterinary Medical Sciences, University of Bologna, Bologna, Italy; ^6^ Myeloproliferative Neoplasm Research Consortium, New York, NY, United States; ^7^ Department of Medicine and Surgery, University Campus Bio-Medico, Rome, Italy

**Keywords:** GATA1, μLCR, erythroid cells, megakaryocytes, hematopoietic progenitor cells

## Abstract

The phenotype of mice carrying the *Gata1* ^*low*^ mutation that decreases expression of *Gata1* in erythroid cells and megakaryocytes, includes anemia, thrombocytopenia, hematopoietic failure in bone marrow and development of extramedullary hematopoiesis in spleen. With age, these mice develop myelofibrosis, a disease sustained by alterations in stem/progenitor cells and megakaryocytes. This study analyzed the capacity of *hGATA1* driven by a *μLCR*/*β-globin* promoter to rescue the phenotype induced by the *Gata1* ^*low*^ mutation in mice. Double *hGATA1*/*Gata1* ^*low/0*^ mice were viable at birth with hematocrits greater than those of their *Gata1* ^*low/0*^ littermates but platelet counts remained lower than normal. *hGATA1* mRNA was expressed by progenitor and erythroid cells from double mutant mice but not by megakaryocytes analyzed in parallel. The erythroid cells from *hGATA1/Gata1* ^*low/0*^ mice expressed greater levels of GATA1 protein and of *α*- and *β*-globin mRNA than cells from *Gata1* ^*low/0*^ littermates and a reduced number of them was in apoptosis. By contrast, *hGATA1/Gata1* ^*low/0*^ megakaryocytes expressed barely detectable levels of GATA1 and their expression of acetylcholinesterase, Von Willebrand factor and platelet factor 4 as well as their morphology remained altered. In comparison with *Gata1* ^*+/0*^ littermates, *Gata1* ^*low/0*^ mice contained significantly lower total and progenitor cell numbers in bone marrow while the number of these cells in spleen was greater than normal. The presence of *hGATA1* greatly increased the total cell number in the bone marrow of *Gata1* ^*low/0*^ mice and, although did not affect the total cell number of the spleen which remained greater than normal, it reduced the frequency of progenitor cells in this organ. The ability of *hGATA1* to rescue the hematopoietic functions of the bone marrow of the double mutants was confirmed by the observation that these mice survive well splenectomy and did not develop myelofibrosis with age. These results indicate that *hGATA1* under the control of *µLCR/β-globin* promoter is expressed in adult progenitors and erythroid cells but not in megakaryocytes rescuing the erythroid but not the megakaryocyte defect induced by the *Gata1* ^*low/0*^ mutation.

## Introduction

GATA1 is a member of the GATA family of transcription factors that ensures appropriate differentiation in hematopoietic cells of multiple lineages including erythroid (Tsai et al., 1989), megakaryocytic (Martin et al., 1990; Romeo et al., 1990), eosinophil (Yu et al., 2002a) and mast cells (Migliaccio et al., 2003). The lineages most stringently controlled by GATA1 are the erythroid and megakaryocytic ones (Orkin and Zon, 2008). In fact, genetically engineered alterations in mice and spontaneous mutations in humans are often associated with X-linked inherited disorders with erythroid and/or megakaryocytic phenotypes (Crispino and Weiss, 2004; Crispino and Horwitz, 2017). In mice, loss of *Gata1* blocks the maturation of erythroid cells at the pro-erythroblast stage inducing the cells into apoptosis (Fujiwara et al., 2002) while selective loss of *Gata1* expression in megakaryocytes (MK) arrests their terminal maturation and retains the cells in proliferation (Shivdasani et al., 1997; Vyas et al., 1999). Therefore, *Gata1* deficient mice die of severe anemia between day 10.5 and 11.5 of gestation with sign of intraembryonic hemorrhage (McDevitt et al., 1997). In human, both inherited and acquired *GATA1* mutations have been described. Point mutations suppressing the binding of the amino-terminal zinc-finger domain of the protein with either the DNA or the nuclear protein FOG1 are found in inherited disorders with erythroid or megakaryocyte deficiency (Nichols et al., 2000; Freson et al., 2001; Mehaffey et al., 2001; Yu et al., 2002b; Di Pierro et al., 2015; Iolascon et al., 2020). The good agreement between the phenotype of the patients and that of mice carrying the corresponding mutations (Campbell et al., 2013; Russo et al., 2017), indicates that these mutations are indeed responsible for sustaining the clinical traits. Based on extensive information indicating that in mice the ratio between the GATA1 and GATA2 content (Gata2/Gata1 switch) controls the proliferation of murine stem/progenitor cells (Bresnick et al., 2010), it is not surprising that acquired frame shift and point mutations impairing GATA1 functions induce leukemia in humans. Mutations resulting in the expression of a shorter amino-truncated protein (GATA1s) are associated with human Acute Megakaryocytic Leukaemia in Down syndrome (DS-AMKL) (Wechsler et al., 2002), transient myeloproliferative syndromes in newborns (Ahmed et al., 2004) and, in rare cases, with adult megakaryocytic leukemia (Harigae et al., 2004). In addition, ribosomopathies affecting the efficiency of translation of *GATA1* mRNA are associated, in addition to Diamond Blackfan Anemia (Ludwig et al., 2014), with primary myelofibrosis, the most severe of the Philadelphia-negative myeloproliferative disorders (Vannucchi et al., 2005; Gilles et al., 2017).

The GATA family genes encode proteins very similar in structure and extremely conserved across species (Trainor et al., 1990). The specificity of their functions is therefore not dictated by the protein structure but rather by their accurate spatial-temporal expression during development and cytogenesis ensured by specific regulatory sequences (Ferreira et al., 2007). As an example, in mice loss of *Gata1* function is rescued by gain of function of either *Gata2* or *Gata3*, providing that their expression is driven by the regulatory sequence of *Gata1* (Ferreira et al., 2005). The regulatory sequences of *Gata1* include three hypersensitive sites which represent putative enhancers (Trainor et al., 1990; Ferreira et al., 2007). Their deletions are defined hypomorphic mutations because they reduce expression and protein content of Gata1 in a lineage-specific fashion. The most studied of these deletions are the *Gata1* ^*0.5*^ developed by the Yamamoto laboratory, that induce a transmissible erythroid leukemia in mice (Shimizu and Yamamoto, 2016), and the *Gata1* ^*low*^ mutation developed by the Orkin laboratory, that induce a lethal anemia and thrombocytopenia at birth in the C57BL6 strain (McDevitt et al., 1997; Vyas et al., 1999) and thrombocytopenia with myelofibrosis in CD1 mice (Vannucchi et al., 2002; Martelli et al., 2005). The phenotype induced by the *Gata1* ^*low*^ mutation in the CD1 background is particularly intriguing. Indeed, these mice are viable at birth because their anemia is rescued within 1 month by recruiting the spleen as extramedullary hematopoietic site (Migliaccio et al., 2009). In this strain, bone marrow hematopoiesis is ineffective as demonstrated by low hematopoietic stem cell content and by the fact that, when splenectomised, these mice die of profound anemia within 15 days (Migliaccio et al., 2009; Spangrude et al., 2016). However, the mice remain thrombocytopenic in spite of the high number of megakaryocytes present in their bone marrow and spleen, and with age develop a syndrome similar to human primary myelofibrosis characterized by fibrosis and hematopoietic failure in bone marrow, and development of hematopoiesis in extramedullary sites (Vannucchi et al., 2002).

To define the function of human GATA1 (*hGATA1*), two transgenic mice lines have been created carrying either the entire *hGATA1* gene or its cDNA under the control of a *μLCR* coupled with the promoter of the *β-globin* gene (Peterson et al., 1993; Li et al., 2002). The line carrying the genomic *hGATA1* dies between 10.5 and 12.5 days of gestation and, in spite of high expression of the transgene in erythroid cells, is anemic. By contrast, the line containing the *μLCR/β-globin* promoter coupled with *hGATA1* cDNA (hereinafter referred to as *hGATA1*) is viable at birth (Li et al., 2002). This observation allowed the generation of *hGATA1*/*β*-YAC doubly hemizygous transgenic mice that were instrumental to demonstrate that *hGATA1* is a specific repressor of the human *ε* gene *in vivo* but is dispensable for the expression of the human *γ* and *β* globin genes (Peterson et al., 1993). However, this demonstration was confounded by the fact that in these mice the *hGATA1* cDNA was expressed at high levels in embryonic and fetal hematopoietic tissues but not in adult tissues (Li et al., 2002). The fact that a following study was capable to detect expression of a LacZ reporter driven by the *μLCR/β-globin* promoter in erythroid cells from adult transgenic mice (Papayannopoulou et al., 2000), raises the possibility that high levels of GATA1 protein, as a consequence of expression of both the *hGATA1* transgene and the endogenous gene, may induce erythroid cells into apoptosis, providing a negative pressure which selects against cells expressing *hGATA1*. This possibility suggested us the hypothesis that the spectrum of cells in which *hGATA1* is expressed is best identified in a genetic contest in which the expression of the endogenous gene is reduced. To test this hypothesis, we analyzed whether *hGATA1* is expressed in hematopoietic cells from adult mice hypomorphic at the *Gata1* ^*low*^ locus, which contain low levels of the endogenous protein in the progenitor, erythroid and megakaryocyte cell compartments. The results obtained indicate that in the context of low GATA1 content, *hGATA1* is expressed in adult erythroid progenitor and precursor cells at levels sufficient to rescue their defective phenotype. By contrast, *hGATA1* was not expressed in cells of the closely related megakaryocyte lineage of these mutants, which remained abnormal, providing evidence that even in the contest of reduced expression of the endogenous gene, the *μLCR/β-globin* promoter is erythroid specific.

## Materials and Methods

### Generation of Double *hGATA/Gata1* ^*low/0*^ Mutants

Transgenic *hGATA1* (*hGATA1/Gata1* ^*+/0*^) males generated as previously described (Li et al., 2002) were crossed with either CD1 (wild type at the *Gata1* locus) or *Gata1* ^*low/low*^ females and their progeny genotyped by polymerase chain reaction (PCR) at birth, as previously described (Li et al., 2002; Vannucchi et al., 2002) (Table 1). Since *Gata1* is on the X chromosome while *μLCR/hGATA1* is autosomal, the offspring of these matings are all heterozygous for *hGATA1* and heterozygous for *Gata1* ^*low*^ when females or hemizygous *Gata1* ^*low/0*^ (wild type) when males. All the experiments were conducted on *Gata1* ^*low/0*^ and *Gata1* ^*+/0*^ males containing or not the *hGATA1* transgene at 3–4 months of age, unless otherwise indicated. Mice were housed under good animal care practice conditions in the animal facilities of Istituto Superiore Sanità and the experiments were performed according to the protocol n. 419/2015-PR approved by the Italian Ministery of Health and according to the directive 2010/63/EU of the European Parliament and of the Council of September 22, 2010 on the protection of animals used for scientific purposes.

**TABLE 1:** Summary of the offsprings of the mating of CD1 or *Gata1* ^*low/low*^ mutants with the *hGATA1* transgenic mice. *hGATA1/Gata1* ^*+/0*^ males were crossed with either CD1 (*Gata1* ^*+/0*^) or *Gata1* ^*low/low*^ females and their progeny genotyped. All the experiments described in the paper were carried out on *Gata1* ^*low/0*^ and *Gata1* ^*+/0*^ males, containing or not the *hGATA1* transgene, highlighted in pink.

	Total number		Genotype of the OFFSPRINGS (%)			
	Litters	Offsprings	♂		♀	
			*Gata1* ^*+/0*^	*hGATA1/Gata1* ^*+/0*^	*Gata1* ^*+/+*^	*hGATA1/Gata1* ^*+/+*^
♀CD1	10	66	23	23	31	23
X						
**♂**						
*hGATA1/Gata1* ^*+/0*^						
			*Gata1* ^*low/0*^	*hGATA1/Gata1* ^*low/0*^	*Gata1* ^*low/+*^	*hGATA1/Gata1* ^*low/+*^
♀*Gata1* ^*low/low*^	20	99	23	38	20	20
X						
♂*hGATA1/Gata1* ^*+/0*^						

### Splenectomy

The spleen was removed from mice anaesthetized with xylazine (10 mg/kg; Bayer) and ketamine (200 mg/kg; Gellini Pharmaceutics) by double ligation of the splenic artery and vein, as described in the animal protocol n. 419/2015-PR. See reference (Migliaccio et al., 2009) for further details.

### Hematological Parameters

Mice were topically anesthetized with lidocaine (one drop/eye) and then blood was collected from the retro-orbital plexus into ethylen-diamino-tetracetic acid-coated microcapillary tubes (20–40 µL/sampling). Hematocrit (Hct) and platelet counts (Ptl) were determined manually.

### Optical, Immunofluorescence and Electron Microscopy Observations

Histological (Hematoxylin and eosin, and Gomori) and Transmission Electron Microscopy (TEM) observations were performed as previously described (Vannucchi et al., 2002; Centurione et al., 2004). For immunofluorescence analysis, spleens were fixed in 10% (v/v) phosphate-buffered formalin, paraffin embedded and cut into 2.5–3 µM sections. Sections were incubated first with an anti-GATA1 monoclonal antibody (M-20, sc-1234, Santa Cruz Biotechnology, Santa Cruz, CA) and then with a fluorescein isothiocyanate (FITC)-conjugated anti-rat IgG antibody (Santa Cruz Biotechnology). After several washes, slides were mounted in glycerol-DABCO containing 4-6-diamidine-2-phenylindole (DAPI, 5 µg/ml) (Sigma-Aldrich, Saint Louis, MO) to counterstain the nuclei. Cells not incubated with the primary antibody served as negative controls. In selected experiments, GATA1 content was evaluated by immune-histochemistry with the same GATA1 antibody followed by peroxidase staining with the DAB CHROMOGEN kit (Bio Optica, Milan, Italy) followed by Hematoxylin staining. Consecutive sections, were instead processed for the terminal deoxy transferase uridine triphosphate nick end-labelling (TUNEL) reaction with the “*In Situ* Cell Death Detection Kit” (Boehringer Mannheim, Mannheim, Germany), as described by the manufacturer. At the end, slides were counter-stained with propidium iodide (Sigma-Aldrich). Samples were analyzed with a light microscope (Leica) equipped with a Coolsnap video camera for computerized images (RS Photometrics, Tucson, AZ, United States).

### Flow Cytometry and Cell Sorting

Mononuclear cells obtained from liver and marrow and light density (*ρ*< 1.080) spleen cells separated over standard Ficoll gradient (Sigma-Aldrich) were suspended in Ca^++^ Mg^++^-free phosphate buffered saline supplemented with 1% (v/v) bovine serum albumin, 2 mM EDTA, 0.1% NaN_3_ and incubated for 30 min on ice with 1 µg/10^6^ cells of phycoerythrin (PE)-conjugated CD117 (anti-cKit), CD71 and CD61, and fluorescein isothiocyanate (FITC)-conjugated anti-CD34, TER119 and CD41 (all from PharMingen, San Diego, CA). Apoptotic cells were detected by double staining with FITC-Annexin V (PharMingen) and propidium iodide (5 μg/ml). In selected experiments, erythroid cells (TER119^pos^/CD71^pos^), megakaryocytes (CD41^pos^/CD61^pos^), hematopoietic stem cell (LSK, lineage negative/CD117^pos^/Sca1^pos^) and erythroid-megakaryocyte (MEP, CD117^pos^/CD34^neg^) and myeloid (CMP/GMP, CD117^pos^/CD34^pos^) restricted progenitor cells were enumerated and eventually prospectively isolated as previously described (Akashi et al., 2000; Ghinassi et al., 2007). The sorted populations were 80–90% pure, upon re-analyses. Cell analysis and sorting were performed using the FACSAria cell sorter (Becton Dickinson, Franklin Lakes, NJ) with three lasers (488-nm argon laser, 599-nm dye laser and ultraviolet laser). Cells labeled with fluorophore-conjugated isotype antibodies (PharMingen) were used to gate non-specific fluorescence signals, while dead cells were excluded on the basis of propidium iodide (5 µg/ml, Sigma) staining.

### Liquid Cultures Under Conditions of Limiting Dilution of Prospectively Isolated Progenitor Cells

MEP cells were cultured under conditions of limiting dilution (3 cells/mL) in 96-well plates containing Iscove modified Dulbecco medium (IMDM; Gibco, Invitrogen, Carlsband, CA) supplemented with fetal calf serum (FCS, 10% v/v; Sigma-Aldrich), 7.5 × 10^−5^ M *β*-mercaptoethanol (Sigma-Aldrich), penicillin, and streptomycin sulfate (50 U/mL, Gibco), and glutamine (2 mM, Gibco). Cultures were stimulated with a cocktail of growth factors contained rat SCF (100 ng/ml; Amgen, Thousand Oaks, CA), murine IL-3 (10 ng/ml, PeproTech, London, United Kingdom) and GM-CSF (10 ng/ml; PeproTech), human FLT3-ligand (10 ng/ml; PeproTech), IL-11 (10 ng/ml; PeproTech) in combination with either thrombopoietin (TPO, 50 ng/ml; PeproTech) (megakaryocyte-permissive conditions) or erythropoietin (EPO, 3 U/mL; Hoffman-La Roche, Basel, Switzerland) (erythroid-permissive conditions), as previously described (Ghinassi et al., 2007). The cultures were incubated at 37°C in a humidified incubator containing 5% CO_2_ in air. Cell growth and phenotype (erythroid: CD71^pos^/TER119^pos^, megakaryocyte: CD117^pos^/CD34^neg^, and mast cells: CD117^pos^/FcεRI^pos^ by flow cytometry) were analyzed 7 days after the beginning of the culture, as described (Ghinassi et al., 2007).

### Ribo Nucleic Acid Isolation and Quantitative Reverse Transcriptase-Polymerase Chain Reaction Analysis

Total RNA was prepared using a commercial guanidine thiocyanate/phenol method (TRIzol, Gibco BRL, Paisley, UK) as described by the manufacturer. Glycogen (20 μg, Roche Ltd., Basel, CH) was added to each sample as a carrier. cDNA was synthesized from 1 μg of total RNA using random primers and SuperScript III (Invitrogen, Life Technologies, Bethesda, MD). Quantitative PCR was carried out as recommended by the manufacture with TaqMan PCR kits specific for the human or the murine *Gata1* gene, or additional erythroid (*Hba-a1* and *Hbb-b1*) or megakaryocytic (*Ache*, *vWF* and *Pf4*) specific genes (Applied Biosystems, Foster City, CA), with the ABI PRISM 7700 Sequence Detection System (Applied Biosystems). For each gene (X) quantitative values were obtained from the threshold cycle number (*C*_tx_), and expressed as 2^−Δ*C*tx^, were Δ*C*_tx_ is the difference between the average *C*_t_ of the target X gene from that of the housekeeping gene (*Gapdh*).

### Statistical Analysis

Statistical analysis was performed by one-way analysis of variance Dunnett’s Multiple Comparison and one-way analysis of variance Tukey Multiple Comparison Test, as indicated. The analyses were performed with GraphPad Prism 5 software for Windows (GraphPad Software, San Diego, California United States).

## Results

### Mendelian Inheritance of the *hGATA1* Transgene

**FIGURE 1:**
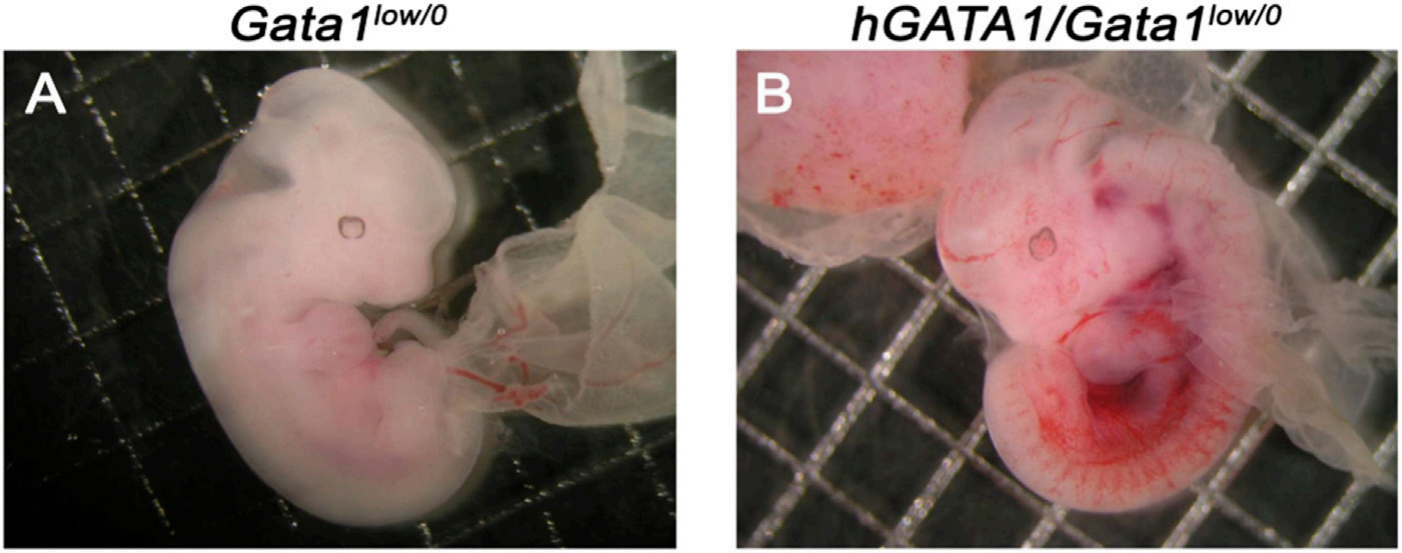
Fetuses from GATA1^*low/0*^ mice carrying the hGATA1 transgene are not anemic. Representative photograph of 11.5 days fetuses from the same litter. The genotype of the fetuses is indicated on the top. Compared to age-matched *Gata1* ^*low/0*^ mice **(A)**, the double mutant *hGATA1/Gata1* ^*low/0*^ mice **(B)** are not anemic. Results are representative of those observed with 5 fetuses per genotype.

Males wild type at the *Gata1* locus (*Gata1* ^*+/0*^) carrying the *hGATA1* transgene (*hGATA1/Gata1* ^*+/0*^) were crossed with either CD1 (wild-type at the *Gata1* locus) or *Gata1* ^*low/low*^ females. For each crossing, 10 and 20 separate matings, respectively, were performed. These crossing generated a total of 66 and 99 pups with an average number of pups per mating of 6.6 and 4.9 for *CD1* and *Gata1* ^*low/low*^ mating, respectively (Table 1). The genotype of the offspring was distributed according to the expected Mendelian ratios. The slight prevalences of wild-type females not carrying *hGATA1* and of *hGATA1*/*Gata1* ^*low/0*^ males are not statistically significant. The Mendelian inheritance of the transgene was associated with a normal phenotype of the embryos (Figure 1). In fact, at both E11.5 and E13.5, *Gata1* ^*low*^ embryos were reported to be pale with clear evidence of anemia (McDevitt et al., 1997). Consistently with this observation, by E11.5 *Gata1* ^*low/0*^ embryos mice were pale, an indication of insufficient red blood cell production (Figure 1A) while E11.5 *hGATA1/Gata1* ^*low/0*^ embryos had a normal morphology, which included a red appearance of the fetal liver (Figure 1B).

### The *hGATA1* Transgene Rescues the Red Cell Counts of the Blood From Adult *Gata1* ^*low*^ Mice

**FIGURE 2:**
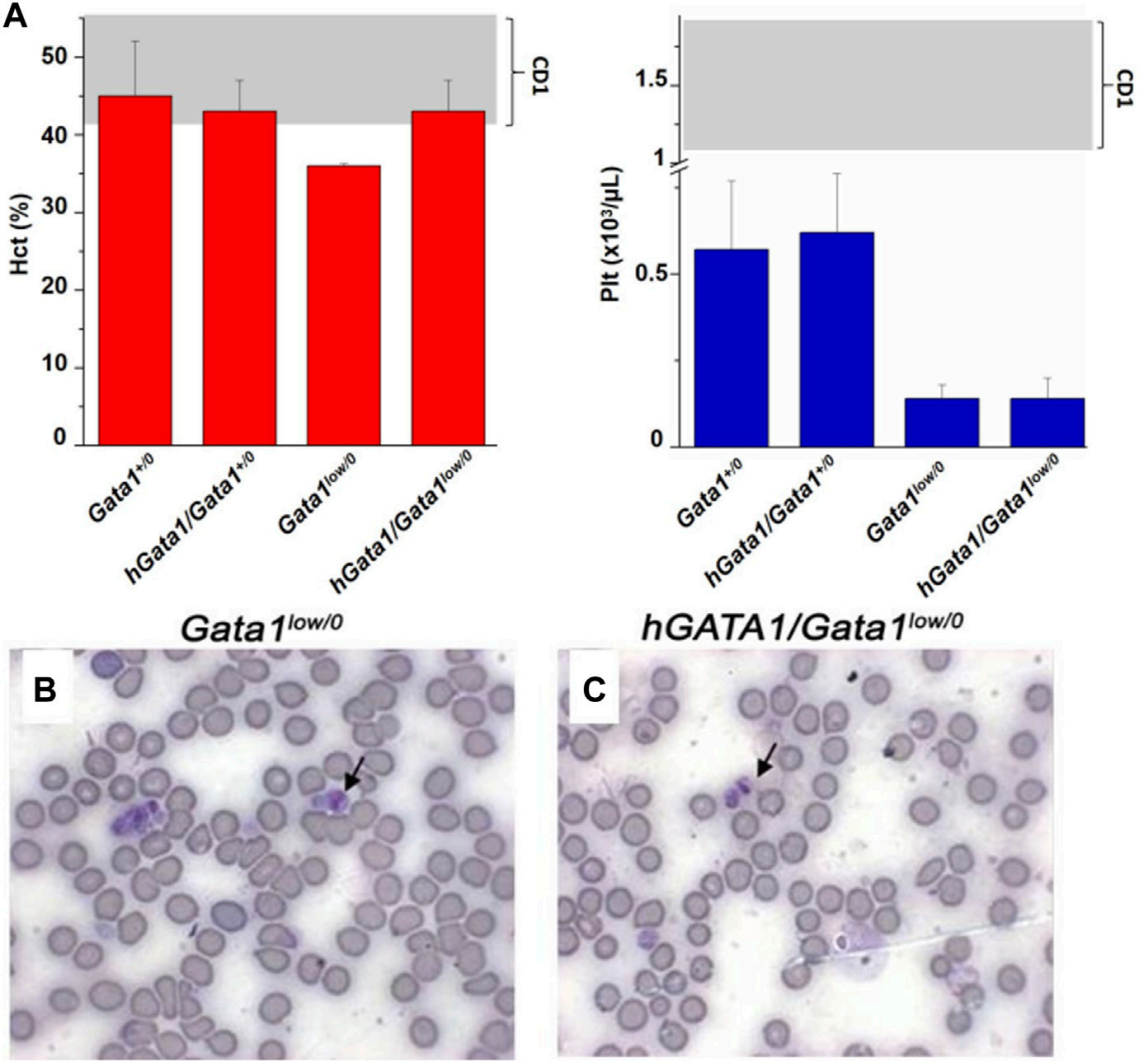
The hGATA1 transgene rescues the hematocrit (Hct) but not the platelet counts of GATA1^*low/0*^ mice. **(A)** Hct and platelet counts of 4 months-old wild-type (*Gata1* ^*+/0*^) mice and *Gata1* ^*low/0*^ mice carrying or not the *hGATA1* transgene, as indicated. The presence of the *hGATA1* transgene restores the Hct of *Gata1* ^*low*^ mice to normal values but does not alter their platelet counts, which remain lower than normal. The shaded areas indicate the range of Hct and Ptl observed in CD1 males available from commercial sources (https://www.criver.com/sites/default/files/resources/CD1IGSMouseModelInformationSheet.pdf). Results are expressed as Mean (±SD) of those obseved with 5 mice per experimental group. Statistical analyses was performed by One-way analysis of variance Tukey Multiple Comparison Test. Results of the statistical analyses: Hct = *Gata1* ^*+/0*^ vs *Gata1* ^*low/0*^ *p* < 0.05, other values are not statistically different; Ptl = *Gata1* ^*+/0*^ vs. *Gata1* ^*low/0*^ *p* < 0.05, *Gata1* ^*+/0*^ vs. *hGATA1/Gata1* ^*low/0*^ *p* < 0.05, *hGata1/Gata1* ^*+/0*^ vs. *Gata1* ^*low/0*^ *p* < 0.05 and *hGata/Gata1* ^*+/0*^ vs. *hGata1/Gata1* ^*low/0*^ *p* < 0.05. **(B,C)** Representative blood smears of the blood from 4 months old *Gata1* ^*low/0*^ **(B)** and *hGATA1/Gata1* ^*low/0*^ **(C)** littermates showing the presence of megathrombocytes in both.

The hematocrit (Hct) and platelet counts of the *Gata1* ^*+/0*^ *, Gata1* ^*low/0*^, *hGATA1/Gata1* ^*+/0*^ and *hGATA/Gata1* ^*low/0*^ mice are compared in Figure 2A. As expected (McDevitt et al., 1997; Vannucchi et al., 2001), *Gata1* ^*low/0*^ males expressed levels of Hct and platelet counts significantly lower than normal. The presence of the *hGATA1* transgene restored the Hct of the *Gata1* ^*low/0*^ mutants up to normal values but had no effects on platelet counts that remained low. This platelet deficiency was confirmed by morphological evaluation that showed reduced number of platelets with size greater than normal (megathrombocytes) in blood smears not only as expected in *Gata1* ^*low/0*^ mice (McDevitt et al., 1997) but also in their *hGATA/Gata1* ^*low/0*^ littermates (Figures 2B,C).

These results indicate that the presence of *hGATA1* rescues the anemia but not the thrombocytopenia induced by the *Gata1* ^*low*^ mutation.

### *hGATA1* is Expressed in Adult Hematopoietic Progenitor and Precursor Cells.

**FIGURE 3:**
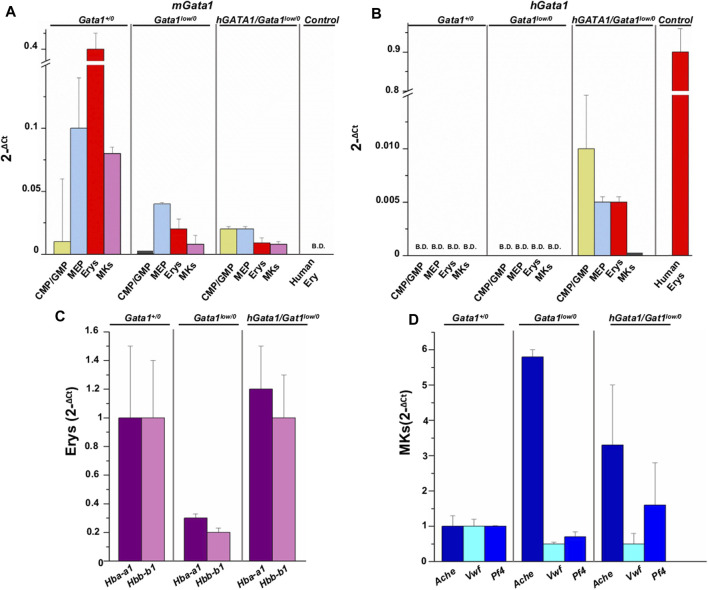
hGATA1 is expressed in erythroid but not in megakaryocyte precursors prospectively isolated from *hGATA1/Gata1* ^*+/0*^ *mice*. Quantitative RT-PCR expression analysis of murine *Gata1* (*mGata1*) **(A)** and *hGATA1* **(B)** mRNA in precursor and progenitor cells prospectively isolated from *Gata1* ^*+/0*^, *Gata1* ^*low/0*^ and *hGATA1*/*Gata1* ^*low/0*^ mice according to the phenotypes: CD117^pos^/CD34^pos^ (CMP/GMP), CD117^pos^/CD34^neg^ (MEP), TER119^pos^/CD71^pos^ (Erys) and CD41^pos^/CD61^pos^ (MKs. The levels of the erythroid specific genes *Hba-a1* and *Hbb-b1* (*α*- and *β*-globin) and of the megakaryocyte specific genes *Ache, Vwf* and *Pf4* (acetylcholinesterase, Von Willebrand factor and platelet factor 4) expressed by the Erys and MKs of the four experimental groups are presented in **(C,D)**, respectively. Data are expressed as 2^−ΔCt^ and are presented as Mean (±SD) of those obseved with cells independently isolated in 3 different experiments each with one mouse per group. *Ex vivo* expanded human erythroblasts (CD235a^pos^/CD36^pos^ cells) served as control for the expression of *hGATA1.* Statistical analyses were performed by One-way analysis of variance Tukey Multiple Comparison Test. Results of the statistical analyses: in **(A,B)**: CMP/GMP: Statistically non-significant; MEP: *Gata1* ^*+/0*^ vs. *Gata1* ^*low/0*^ *p* < 0.05; *Gata1* ^*+/0*^ vs. *hGATA1/Gata1* ^*low*/0^ *p* < 0.01; Erys: Statistically non-significant; MKs: *Gata1* ^*+/0*^ vs. *Gata1* ^*low/0*^ *p* < 0.001 and *Gata1* ^*+/0*^ vs. *hGATA1/Gata1l* ^*ow/0*^ *p* < 0.001. In **(C,D)**: Erys = *Hba-a1*: *Gata1* ^*low/0*^ vs. *hGATA1/Gata1* ^*low/0*^ *p* < 0.05; *Hbb-b1*: *Gata1* ^*+/0*^ vs. *Gata1* ^*low/0*^ and *Gata1* ^*low/0*^ vs. *hGATA1/Gata1* ^*low/0*^ *p* < 0.05; MKs = *Ache*: *Gata1* ^*+/0*^ vs. *Gata1* ^*low/0*^ *p* < 0.01; *Vwf*: *Gata1* ^*+/0*^ vs. *Gata1* ^*low/0*^ *p* < 0.01; *Gata1* ^*+/0*^ vs. *hGATA1/Gata1* ^*low/0*^ *p* < 0.01.

By quantitative RT-PCR, we compared the levels of *hGATA1* and of the endogenous murine *Gata1* (*mGata1*) gene expressed by adult hematopoietic progenitor and precursors cells prospectively isolated from the bone marrow of adult *Gata1* ^*+/0*^, *Gata1* ^*low/0*^ and *hGATA1*/*Gata1* ^*low/0*^ mice (Figure 3). Human erythroblasts (CD235^pos^/CD36^pos^ cells), expanded *in vitro* as previously described (Federici et al., 2019), were analyzed in parallel as positive control for *hGATA1* expression. We measured only the total level of *Gata1* mRNA because in humans the ribosomopathy induced by the driver mutations found in myelofibrosis equally reduces the megakaryocyte content of the full-length and short isoform of the GATA1 protein (Vannucchi et al., 2005; Gilles et al., 2017).

As expected (Ling et al., 2018), CMP (by ∼25-fold), MEP (by ∼2.5-fold), erythroid precursors (by ∼20-fold) and megakaryocytes (by ∼10-fold) from *Gata1* ^*low/0*^ mice expressed significantly lower levels of *mGata1* mRNA than the corresponding cells from their *Gata1* ^*+/0*^ littermates. The presence of *hGATA1* did not affect expression of the endogenous gene in these populations purified from *hGATA1*/*Gata1* ^*low/0*^ mice, which remained lower than normal (Figure 3).

Unsurprisingly, *hGATA1* was not detected in cells prospectively isolated from *Gata1* ^*+/0*^ or *Gata1* ^*low/0*^ littermates but it was expressed already at the level of progenitor cells (both CMP and MEP) prospectively isolated from *hGATA1*/*Gata1* ^*low/0*^ mice. In these mice, expression of *hGATA1* persisted at the level of the erythroid precursors while it was barely detectable in megakaryocytes (Figure 3).

These results indicate that in the *Gata1* ^*low/0*^ genetic contest, the expression of *hGATA1* driven by the *μLCR* coupled with the *β-globin* gene promoter is restricted to progenitor and erythroid cells and that its expression does not affect the levels of the endogenous gene which remain lower than normal.

### *hGATA1* Rescues the Defective Phenotype of Erythroid Cells but not That of Megakaryocytes from *Gata1* ^*low/0*^ Mice.

**FIGURE 4:**
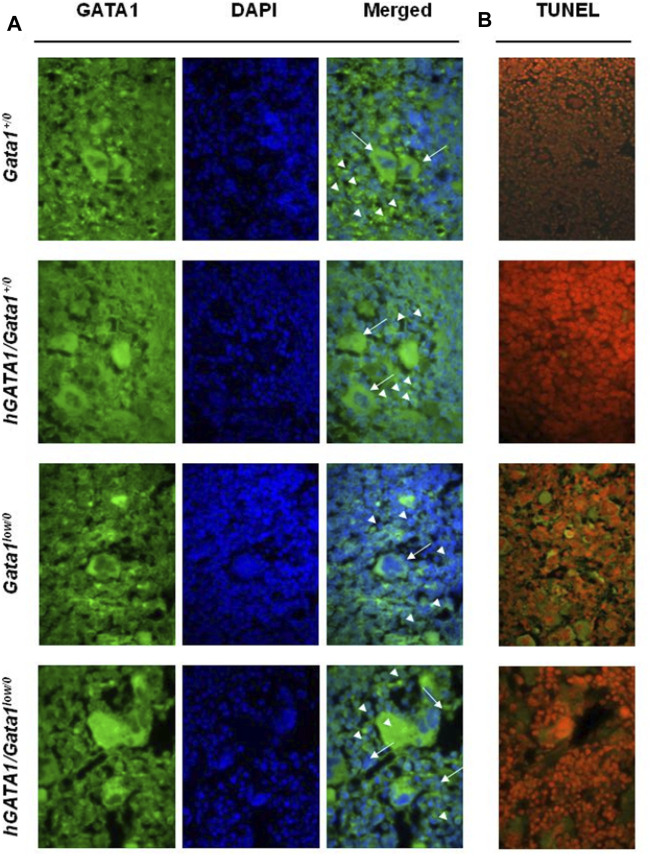
Erythroid cells from adult hGATA1/GATA1^*low/0*^ double mutants contain high levels of GATA1 protein and express low apoptotic rates. **(A)** Representative confocal microscopy analysis of GATA1 content of spleen sections from wild-type and *Gata1* ^*low/0*^ mice carrying or not the *hGATA1* transgene, as indicated. Arrowheads and arrows indicate representative erythroid and megakaryocytes cells, respectively. GATA1 content (green fluorescence) increases in the nuclei of erythroid cells from *hGATA1/Gata1* ^*low/0*^ mice whereas remains low in their megakaryocytes. **(B)** Confocal microscopy analysis by TUNEL assay indicates reduced number of apoptotic cells in the spleen from *hGATA1/Gata1* ^*low/0*^ compared to those present in *Gata1* ^*low/0*^ mice. Results are representative of those observed with 3 mice per experimental groups. Similar results were observed by immunostaining with 2 additional mice per experimental groups (Supplementary Figure S1A). Original Magnification 64X.

In accordance to the *Gata1* ^*low*^ levels detected by RT-PCR (Figure 3), by immunofluorescence, erythroid cells in the spleen from *Gata1* ^*low/0*^ mice contain low levels of GATA1 protein (Figure 4A). By contrast, the erythroid cells in the spleen from mice containing the *hGATA1* transgene were more intensively green fluorescent indicating a higher GATA1 content. The GATA1 protein is synthesized in the cytoplasm and translocate in the nucleus as part of the NuRSERY complex formed by HADC5, GATA1, EKLF and ERK in response to factors that phosphorylate ERK (Varricchio et al., 2014). It is not surprising, then, that the GATA1 fluorescent signal was detected both in the cytoplasm and in the nucleus of the cells (see also Supplementary Figure S1). Of note, the fluorescence intensity is particularly evident in the nuclei of the *hGATA1/Gata1* ^*low/0*^ mutants. The antibody used to detect GATA1 does not discriminate between the murine and the human protein. Since the levels of the endogenous *mGata1* expressed by erythroid cells from *Gata1* ^*low/0*^ and *hGATA1/Gata1* ^*low/0*^ mice is the same (Figure 3), we believe that the greater levels of GATA1 protein detected in the erythroid cells from the double mutants is the result of *hGATA1* expression.

Despite extramedullary hematopoiesis in the spleen corrects erythropoiesis of *Gata1* ^*low*^ mice (Migliaccio et al., 2009), these animals remain anemic due to the increased apoptotic rates of their erythroid cells, detected by TUNEL staining of this organ (Vannucchi et al., 2001). In order to assess whether the greater GATA1 content of the erythroid cells from the spleen of double mutants rescued their increased apoptosis rates, TUNEL staining was performed. As expected, TUNEL staining was limited in the spleen from wild type mice but it greatly increased in the erythroblasts from the spleen of *Gata1* ^*low/0*^ mutants (Figure 4B). Since the localization of the TUNEL staining is expected to be perinuclear (Vannucchi et al., 2001 and Supplementary Figure S1B), it is not surprising that at the magnification presented in Figure 4B the Tunnel signal is observed in the cytoplasm.

By contrast, the presence of the *hGATA1* transgene drastically reduced the frequency of TUNEL-positive cells present in spleen sections from the double mutant mice (Figure 3B). These results were confirmed at the bone marrow level by flow cytometry determinations showing that erythroid cells from double mutant mice, by contrast with those from their *Gata1* ^*low/0*^ counterpart, are barely stained by Annexin V. In fact, the frequency of Annexin^pos^ Ter119^pos^ cells in the bone marrow was 25.58 ± 13.8 (*n* = 4) in *Gata1* ^*low/0*^ mice vs. 4.17 ± 5.22 in *hGATA1Gata1* ^*low/0*^ mice (*n* = 6) (*p* = 0.0078 by ANOVA) while the frequency of Annexin^pos^ Ter119^pos^ cells in the bone marrow from two *Gata1* ^*+/0*^ mice analysed in parallel was 0.5 and 0.7.

In agreement with the observation that *hGATA1* mRNA was similarly undetectable in megakaryocytes from *Gata1* ^*low/0*^ and *hGATA1/Gata1* ^*low/0*^ littermates (Figure 3), immunofluorescence determinations indicated that the GATA1 protein was barely detectable in the megakaryocytes from the spleen of these experimental groups (Figure 4A and Supplementary Material S1A).

To confirm that *hGATA1* rescues erythropoiesis in the bone marrow of *Gata1* ^*low/0*^ mice, the expression of specific erythroid and megakaryocytic genes was assessed in erythroid cells and megakaryocytes prospectively isolated from the bone marrow of *Gata1* ^*+/0*^, *Gata1* ^*low/0*^ and *hGATA1*/*Gata1* ^*low/0*^ mice (Ghinassi et al., 2007). Compared to wild type mice, erythroid cells from *Gata1* ^*low/0*^ mice expressed significantly lower levels of *α*- and *β*-globins, that were restored up to normal levels in cells from the double mutant (Figure 3C). By contrast, in megakaryocytes the presence of *hGATA1* transgene did not affect the expression of acetylcholinesterase, Von Willebrand factor, and platelet factor 4 in *hGATA1*/*Gata1* ^*low/0*^ mice which remained similar to that detected in cells from *Gata1* ^*low/0*^ mice and abnormally greater than that of cells from *Gata1* ^*+/0*^ littermates (Figure 3D).

In conclusion, the observation that erythroid cells but not the megakaryocytes from the double mutant mice contain levels of GATA1 protein similar to normal provides a mechanistic insight to explain why the presence of *hGATA1* rescues the abnormal maturation of erythroid cells but not that of megakaryocytes induced by the *Gata1* ^*low*^ mutation.

### The *hGATA1* Transgene Induces Extramedullary Hematopoiesis in Both Spleen and Liver

**FIGURE 5:**
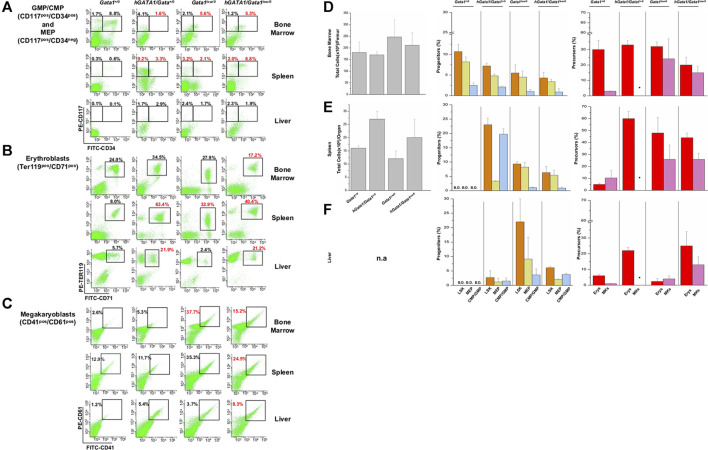
The presence of hGATA1 increases the frequency of hematopoietic cells in the bone marrow and liver of *Gata1* ^*low/0*^ mice and in the liver of wild type (Gata1+/0) mice. **(A–C)** Gates used to identify GMP/CMP and MEP, erythroblasts and megakaryocytes in organs from representative *Gata1* ^*+/0*^, *hGATA1/Gata1* ^*+/0*^, *Gata1* ^*low/0*^ and *hGATA1/Gata1* ^*low/0*^ mice are indicated in panel **(A–C)**, respectively. The numbers on the top of the gates indicate the frequency of the cell population in that particular experiment. **(D–F)** Total cell numbers (left panels) and frequency of stem/progenitors cells (LSK, Lin^−^/CD117^pos^/Sca1^pos^, CMP/GMP, CD117^pos^/CD34^pos^, MEP, CD117^pos^/CD34^neg^) (middle panels) and of erythroid (Erys, TER119^pos^/CD71^pos^) and megakaryocyte (MKs, CD41^pos^/CD61^pos^) precursors (right panels) in the femur **(D)**, spleen **(E)** and liver **(F)** from *Gata1* ^*+/0*^, *hGATA1/Gata1* ^*+/0*^, *Gata1* ^*low/0*^ and *hGATA1/Gata1* ^*low/0*^ mice. Results are expressed as Mean (±SD) of those obseved with 3 mice per experimental group. Statistical analyses was performed by One-way analysis of variance Tukey Multiple Comparison Test. Results of the statistical analyses: Bone marrow = total cell number: *Gata1* ^*+/0*^ vs *hGATA1/Gata1* ^*+/0*^ *p* < 0.05 and *hGATA1/Gata1* ^*+/0*^ vs. *Gata1* ^*low/0*^ *p* < 0.01; LSK: *Gata1* ^*+/0*^ vs. *Gata1* ^*low/0*^ *p* < 0.05 and *Gata1* ^*+/0*^ vs. *hGATA1/Gata1* ^*+/0*^ *p* < 0.01; CMP/GMP: *Gata1* ^*+/0*^ vs. *hGATA1/Gata1* ^*+/0*^ *p* < 0.05 and *Gata1* ^*+/0*^ vs. *Gata1* ^*low/0*^ *p* < 0.05 and Gata1+/0 vs. *hGATA1/Gata1* ^*low/0*^ *p* < 0.01; MEP: *Gata1* ^*+/0*^ vs. *Gata1* ^*low/0*^ *p* < 0.05 and *Gata1* ^*+/0*^ vs. *hGATA1/Gata1* ^*low/0*^ *p* < 0.05; Erys: *hGATA1/Gata1* ^*+/0*^ vs. *hGATA1/Gata1* ^*low/0*^ *p* < 0.05 and *Gata1* ^*low/0*^ vs. *hGATA1/Gata1* ^*low/0*^ *p* < 0.05, MKs: *Gata1* ^*+/0*^ vs. *Gata1* ^*low/0*^ *p* < 0.05. Spleen = total cell number: Statistically non-significant; LSK: *Gata1* ^*+/0*^ vs. *hGATA1/Gata* ^*1+/0*^ *p* < 0.001, *Gata1* ^*+/0*^ vs. *Gata1* ^*low/0*^ *p* < 0.001, *Gata1* ^*+/0*^ vs. *hGATA1/Gata1* ^*low/0*^ *p* < 0.01, *hGATA1/Gata1* ^*+/0*^ vs. *Gata1* ^*low/0*^ *p* < 0.001 and *hGATA1/Gata1* ^*+/0*^ vs. *hGATA1/Gata1* ^*low/0*^ *p* < 0.001; CMP/GMP: *Gata1* ^*+/0*^ vs. *hGATA1/Gata1* ^*+/0*^ *p* < 0.05, *Gata1* ^*+/0*^ vs. *Gata1* ^*low/0*^ *p* < 0.001, *Gata1* ^*+/0*^ vs. *hGATA1/Gata1* ^*low/0*^ *p* < 0.01 and *hGATA1/Gata1* ^*+/0*^ vs. *Gata1* ^*low/0*^ *p* < 0.01; MEP: *Gata1* ^*+/0*^ vs. *hGATA1/Gata1* ^*+/0*^ *p* < 0.001, *hGATA1/Gata1* ^*+/0*^ vs. *Gata1* ^*low/0*^ *p* < 0.001 and *hGATA1/Gata1* ^*+/0*^ vs. *hGATA1/Gata1* ^*low/0*^ *p* < 0.001; Erys: *Gata1* ^*+/0*^ vs. *hGATA1/Gata1* ^*+/0*^ *p* < 0.001, *Gata1* ^*+/0*^ vs. *Gata1* ^*low/0*^ *p* < 0.001 and *Gata1* ^*+/0*^ vs. *hGATA1/Gata1* ^*low/0*^ *p* < 0.001; MKs: Statistically non-significant. Liver = LSK: *Gata1* ^*+/0*^ vs. *hGATA1/Gata1* ^*low/0*^ *p* < 0.01, *hGATA1/Gata1* ^*+/0*^ vs. *hGATA1/Gata1* ^*low/0*^ *p* < 0.05 and *Gata1* ^*low/0*^ vs *hGATA1/Gata1* ^*low/0*^ *p* < 0.05; CMP/GMP: Statistically non-significant; MEP: Statistically non-significant; Erys: *Gata1* ^*+/0*^ vs. *hGATA1/Gata1* ^*+/0*^ *p* < 0.05, *Gata1* ^*+/0*^ vs. *hGATA1/Gata1* ^*low/0*^ *p* < 0.01, *hGATA1/Gata1* ^*+/0*^ vs. *Gata1* ^*low/0*^ *p* < 0.01 and *Gata1* ^*low/0*^ vs. *hGATA1/Gata1* ^*low/0*^ *p* < 0.01; MKs: *Gata1* ^*+/0*^ vs. *hGATA1/Gata1* ^*low/0*^ *p* < 0.01 and *Gata1* ^*low/0*^ vs. *hGATA1/Gata1* ^*low/0*^ *p* < 0.01.

In *Gata1* ^*low*^ mice hematopoiesis fails in the bone marrow and is active in the spleen which contains most of the hematopoietic stem cells of these animals (Spangrude et al., 2016). To assess the effect of the *hGATA1* transgene on the active site of hematopoiesis, total cell number and frequency of hematopoietic stem/progenitor and precursors cells in bone marrow, spleen and liver from *Gata1* ^*+/0*^, *hGATA1*/*Gata1* ^*+/0*^, *Gata1* ^*low/0*^ and *hGATA1*/*Gata1* ^*low/0*^ mice were compared (Figure 5 **)**. Hematopoietic stem/progenitor cells are defined as Lin^−^/CD117^pos^/Sca1^pos^ cells (LSK) and divided by CD34 staining into bipotent erythro-megakaryocytic (MEP, CD34^neg^) and common myeloid/bipotent granulo-monocytic (CMP/GMP, CD34^pos^) progenitor cells (Akashi et al., 2000; Ghinassi et al., 2007).

Bone marrow. As previously reported (Vannucchi et al., 2002), the bone marrow from *Gata1* ^*low/0*^ mice contained statistically significant lower numbers of cells than that from wild type mice (Figure 5). Significantly lower than normal were also the frequencies of GMP/CMP while the slightly lower frequency of MEP was not statistically significant. At the precursor level, the frequency of erythroid cells was within normal values while that of megakaryocytes, as expected (Vannucchi et al., 2002), was greater than normal. The presence of *hGATA1* had a small not significant impact on the cellularity of the femur from *Gata1* ^*low/0*^ animals. However, it significantly decreased the frequency of erythroid precursors in this organ (a sign of increased maturation) and had no effect on the frequency of progenitor cells and megakaryocytes that remained, respectively, lower and higher than normal (Figure 5).

Spleen. As reported (Vannucchi et al., 2002), the total cell number of the spleen from *Gata1* ^*low/0*^ mice was about 0.4-fold greater than normal (Figure 5). However, due to great variability in this organ size among individual mice, there was insufficient power to demonstrate statistically significant differences with the low number (three) of mice analyzed per group. The spleen from these mice contained greater frequencies of progenitor and precursor cells than that of hemizygote *Gata1* ^*+/0*^ mice in which these cell populations were barely detectable. The presence of *hGATA1* dramatically increased the frequency of LSK and progenitor cells and that of erythroid precursors in the spleen from *Gata1* ^*+/0*^ mice. However, although the frequency of these cell populations in the spleen from *hGATA1/Gata1* ^*low/0*^ remained greater than that of wild type mice, it was significantly lower when compared to that of *hGATA1/Gata1* ^*+/0*^ mice (Figure 5).

Liver. Although the total number of cells in the liver of the various mice groups was not recorded, the liver from transgenic mice containing *hGATA1* appeared pale, an indication of reduced vascularization, and with a gummy consistency, an indication of altered tissue architecture (data not shown). In agreement with these observations, the liver from mice containing the human transgene, *hGATA1/Gata1* ^*+/0*^ and *hGATA1*/*Gata1* ^*low/0*^ alike, contained detectable numbers of LSK and hematopoietic progenitors, and significantly higher numbers of erythroid and megakaryocyte precursors compared to wild type mice (Figure 5). Of note, although the liver from *Gata1* ^*low/0*^ mice contained the greatest number of progenitor cells, the frequency of the erythroid and megakaryocyte precursors in this organ is very low, confirming that the liver is not an active hematopoietic site in *Gata1* ^*low/0*^ mice at least at the age analyzed (4-months of age) (Vannucchi et al., 2002).

These results indicate that the presence of *hGATA1* induces hematopoiesis in the bone marrow and reduces that in the spleen in mice carrying the *Gata1* ^*low*^ mutation.

### *hGATA1* Rescues the Altered Differentiation Potential Expressed *in vitro* by Erythroid-Megakaryocyte Carrying the *Gata1* ^*low*^ Mutation

**FIGURE 6:**
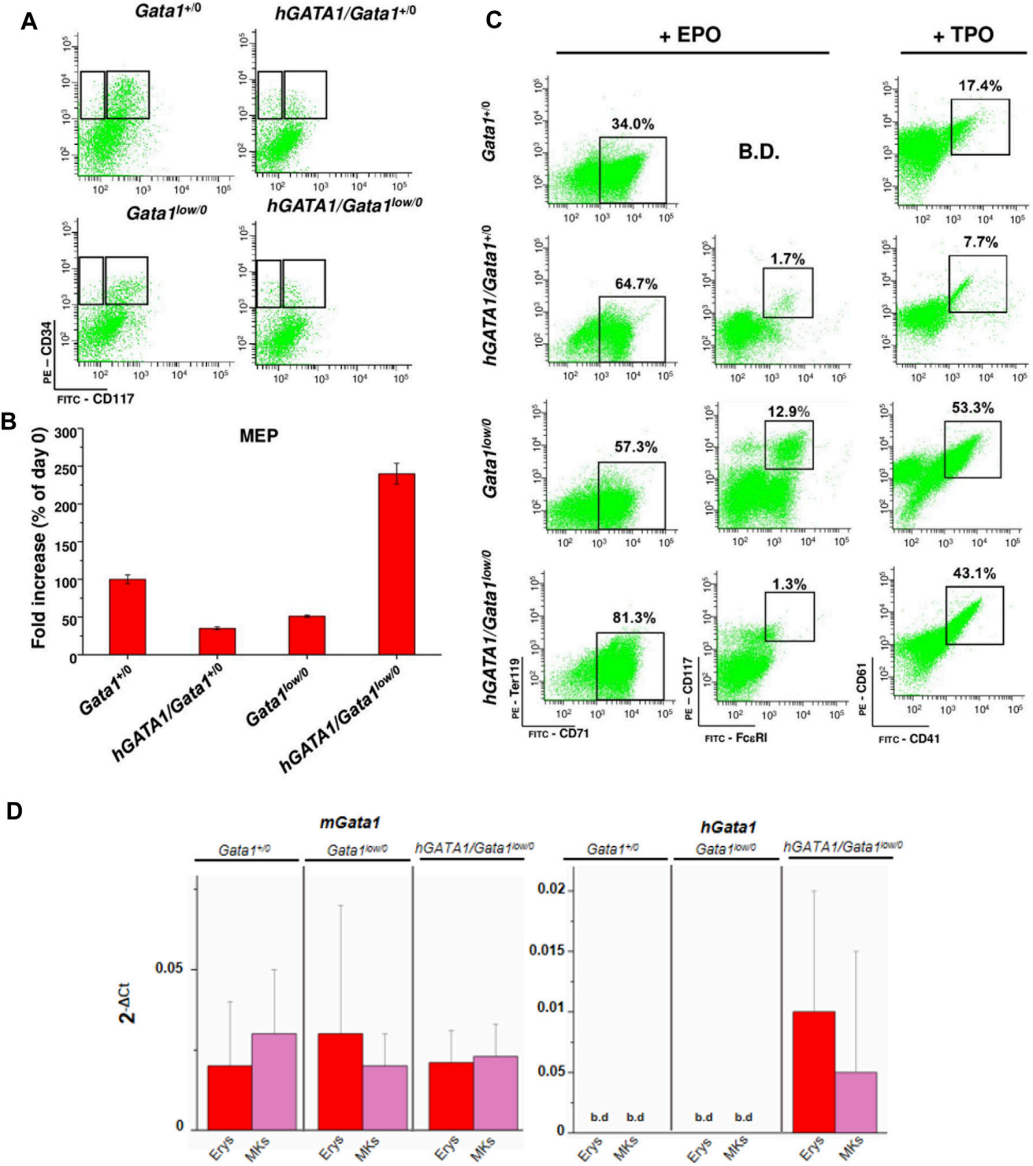
The presence of *hGATA1* reduced number of mast cells generated by MEP carrying the *Gata1* ^low^ mutation *in vitro*, rescuing their abnormal maturation potential. **(A)** Representative gates used for the prospective purification of MEP (right gate) and CMP/GMP (left gate) progenitor cells from *Gata1* ^*+/0*^, *hGATA1/Gata1* ^*+/0*^, *Gata1* ^*low/0*^ and *hGATA1/Gata1* ^*low/0*^ mice, as indicated. **(B)** Fold increase by day 7 in cultures of MEP prospectively isolated from the different animal groups. Since the fold increases observed under erythroid and megakaryocytes culture conditions were the same, results obtained under the two conditions were pooled. **(C)** Analysis of the phenotype expressed by the progeny generated by MEP after 7 days of liquid culture under erythroid- or megakaryocyte-permissive conditions, as indicated. Erythroid, mast cells and megakaryocytes were identified according to the TER119^pos^/CD71^pos^, CD117^pos^/FcεRI^pos^ and CD41^pos^/CD61^pos^ phenotype, respectively, as described (Ghinassi et al., 2007). B.D.: below detection. **(D)** Quantitative RT-PCR expression analysis of *hGATA1* and murine *Gata1* (*mGata1*) mRNA in the erythroid (Erys) and megakaryocyte (MKs) progeny of MEP prospectively isolated from *Gata1* ^*+/0*^, *Gata1* ^*low/0*^ and *hGATA1*/*Gata1* ^*low/0*^ mice. Results are presented as Mean (±SD) of at least three experiments, each one with one mouse per experimental group. Statistical analyses were performed by One-way analysis of variance Dunnett’s Multiple Comparison Test. Results of the statistical analyses: in B and D there are no statistically significant differences among groups.

In a previous publication, we demonstrated that the *Gata1* ^*low*^ mutation increases the proliferation potential of MEP which acquire at the single cell level the ability to generate *in vitro*, in addition to erythroid cells and megakaryocytes, also mast cells with the phenotype CD117^pos^/FcεRI^pos^ (Ghinassi et al., 2007). To clarify whether the expression of the transgene rescues the differentiation potential of *Gata1* ^*low/0*^ MEP, the cells were prospectively isolated from bone marrow of *Gata1* ^*+/0*^, *hGATA1*/*Gata1* ^*+/0*^, *Gata1* ^*low/0*^ and *hGATA1*/*Gata1* ^*low/0*^ mice using gates described in Figure 6A, and cultured under condition of limiting dilutions stimulated with either erythroid- or megakaryocyte-permissive growth factors. The number and phenotype of their progeny were then analyzed after 7 days of culture (Figures 6B,C). MEP from wild type mice generated by day 7 cells with a fold increase of about 100. Approximately 34 and 17% of their progeny were erythroid cells (TER119^pos^/CD71^pos^) and megakaryocytes (CD41^pos^/CD61^pos^), respectively (Figures 6B,C). Although, MEP from *hGATA1*/*Gata1* ^*+/0*^ mice generated a lower number of cells (fold increase ∼50), the great majority of their progeny was erythroid, with low numbers of megakaryocytes even when the cells were cultured under megakaryocyte-specific conditions, and very few mast cells (CD117^pos^/FcεRI^pos^) (Figures 6B,C). As expected, also MEP carrying the *Gata1* ^*low/0*^ mutation expressed a fold increase of ∼50%. However, the progeny of these MEP contained not only great numbers of erythroid cells, but also mast cells and megakaryocytes (Figures 6B,C), confirming that this mutation alters the differentiation potential of these cells (Ghinassi et al., 2007). The fact that the MEP were cultured under conditions of limiting dilution indicate that this abnormality is cell autonomous. By contrast, MEP from *hGATA1Gata1* ^*low/0*^ mice, generated by day 7 significantly more cells than those isolated from the other groups (fold increase ∼250). By contrast with the progeny of *Gata1* ^*low*^ MEP, that of double mutant MEP contained mostly erythroid cells, with very few mast cells, in cultures stimulated with erythroid specific conditions while the frequency of megakaryocytes in cultures stimulated with megakaryocyte-specific growth factors remained high (Figures 6B,C).

Mechanistic insights for these results were provided by assessment of the expression of *mGata1* and *hGATA1* mRNA in erythroid precursors and megakaryocytes generated *in vitro* from MEP cells prospectively isolated from *Gata1* ^*+/0*^, *Gata1* ^*low/0*^ and *hGATA1*/*Gata1* ^*low/0*^ mice (Figure 6D). Once again, *hGATA1* was detected only in the progeny of the double mutant mice and the expression of this gene was greater in erythroid that in megakaryocyte precursors. The fact that, by contrast with the primary megakaryocytes, megakaryocytes expanded *in vitro* expressed detectable levels of *hGATA1* may reflect functional differences between the two populations (Abbonate et al., 2016; Malara et al., 2014).

These results indicate that *hGATA1* rescued the abnormal differentiation potential of *Gata1* ^*low*^ MEP making them unable to generate mast cells in culture.

### The *hGATA1* Transgene Rescues Erythroid Failure in Bone Marrow of *Gata1* ^*low/0*^ Mice

In a previous publication, we had shown that *Gata1* ^*low*^ mice die within 15 days from removal of the spleen, an indication that in these mice erythropoiesis fails in the bone marrow and is sustained mainly by the spleen (Migliaccio et al., 2009). To assess whether the greater levels of GATA1 content in the erythroid cells from *Gata1* ^*low/0*^ mice carrying the *hGATA1* transgene rescue the erythropoietic functions of the marrow of these animals, we compared survival rates of untreated *hGATA1*/*Gata1* ^*low/0*^ littermates with those subjected to splenectomy. To take into account that the functions of the bone marrow from *Gata1* ^*low/0*^ mice are particularly reduced after 8-months of age when the mutant develop myelofibrosis (Vannucchi et al., 2002), experiments were repeated with 8- and 14-months old mice. Splenectomized *Gata1* ^*low/0*^ mice were analyzed as positive controls.

**FIGURE 7:**
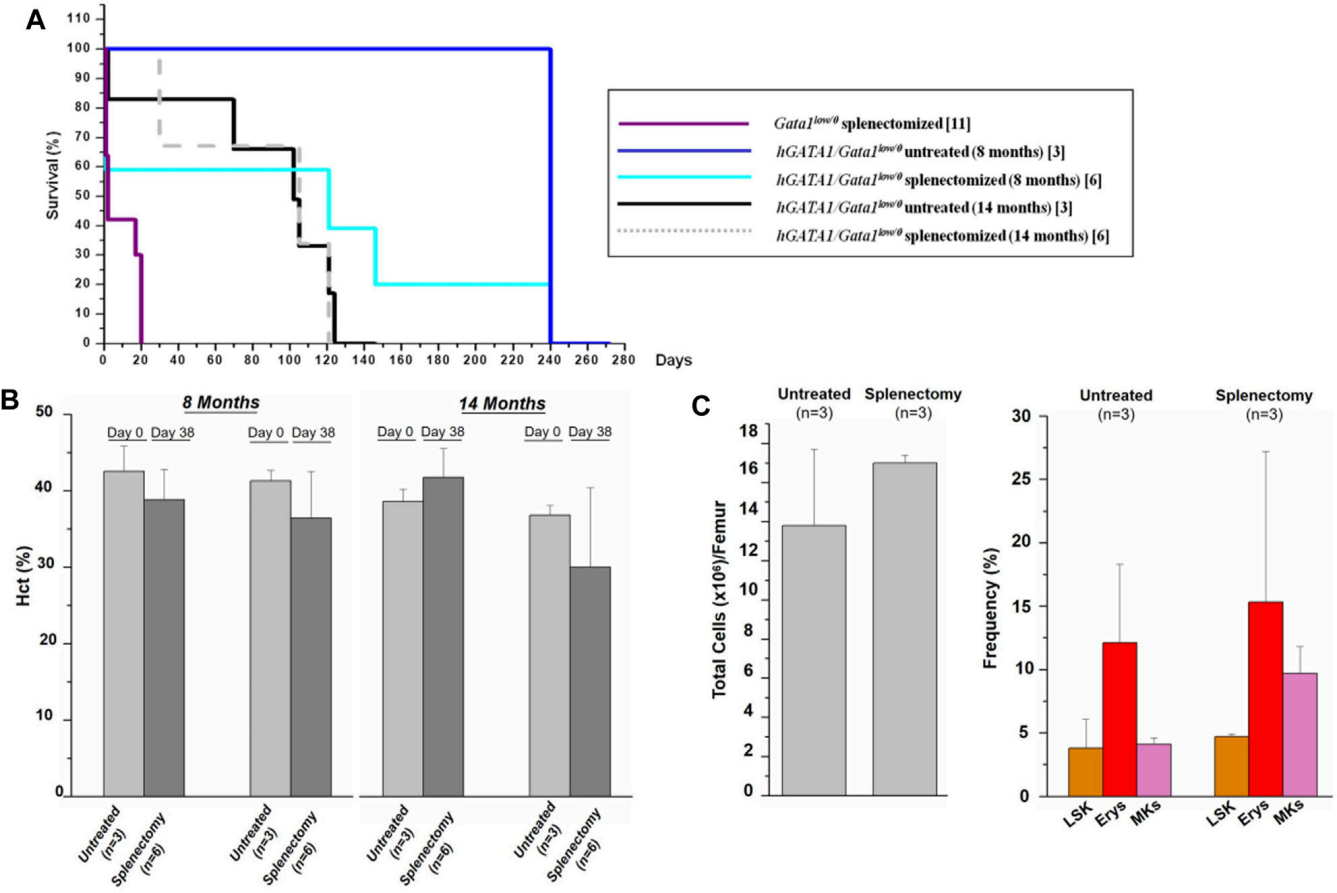
The presence of *hGATA1* rescues erythropoiesis in the bone marrow of *Gata1* ^*low/0*^ mice. Survival **(A)** and hematocrit **(B)** of untreated *hGATA1/Gata1* ^*low/0*^ mice and of their littermates subjected to splenectomy. At the time of splenectomy, mice were either 8- or 14-month old, as indicated. In comparison to *Gata1* ^*low/0*^ mice, *hGATA1/Gata1* ^*low/0*^ survive well splenectomy, demonstrating that the bone marrow in these mice is a functional erythroid organ. Furthermore, at day 38, untreated and splenectomized mice have similar Hct values and these values are not different from those observed at day 0, demonstrating that the spleen has a marginal role in the erythropoiesis of *Gata1* ^*low/0*^ mice carrying the *hGATA1* transgene. **(C)** Total cell number (left panel) and frequency of LSK (Lin^−^/CD117^pos^/Sca1^pos^), and erythroid (TER119^pos^/CD71^pos^) and megakaryocyte (CD41^pos^/CD61^pos^) precursors (right panel) from untreated *hGATA1/Gata1* ^*low/0*^ mice and from their littermates subjected to splenectomy. The mice were splenectomized at 8-months of age and analyzed 240 days after the surgery. (*n*) indicates the number of mice included in each experimental group. Statistical analyses was performed by One-way analysis of variance Dunnett’s Multiple Comparison Test. Results of the statistical analyses: in **(B,C)** there are no statistically significant differences among groups.

As expected (Migliaccio et al., 2009), all *Gata1* ^*low/0*^ mice die within 15–20 days from splenectomy (Figure 7A), confirming the fundamental role of this organ in sustaining their erythropoiesis. By contrast, splenectomy did not affect survival and Hct values of *hGATA1*/*Gata1* ^*low/0*^ mice (Figures 7A,B). In addition, no significant differences were detected in the cellularity and progenitor/precursor cell frequency in the bone marrow from *hGATA1*/*Gata1* ^*low/0*^ splenectomized mice in comparison to that of untreated littermates (Figure 7C).

Collectively, these data provide functional support for the conclusion that *hGATA1* rescues the erythroid failure of the bone marrow of *Gata1* ^*low/0*^ mice.

### Although *hGATA1* Does not Rescue the Abnormal Maturation of *Gata1* ^*low*^ Megakaryocytes, it Reduces the Bone Marrow Fibrosis Developed by the *Gata1* ^low^ Animal Model

Several lines of evidence in patients (Vannucchi et al., 2005; Gilles et al., 2017) and in mouse models, including the *Gata1* ^*low*^ model (Vannucchi et al., 2002; Malara et al., 2018) suggest that myelofibrosis, a disease associated with hematopoietic failure in the bone marrow and development of hematopoiesis in extramedullary sites, is sustained by proinflammatory cytokines secreted at high levels by immature megakaryocytes, a cellular hallmark of this disorder. However, both in patients (Vainchenker et al., 2019) and in *Gata1* ^*low*^ mice (Ghinassi et al., 2007), the disease is also associated with increased proliferation of the stem/progenitor cells sustained by the driver mutations. The observation that the presence of *hGATA1* rescued erythropoiesis in the bone marrow (Figure 5) and normalized the proliferation potential of the MEP (Figure 6) but did not restore the maturation of the megakaryocytes (Figures 2, 3) suggested to us to assess the relative importance of these two abnormalities for the development of the disease by analyzing whether *hGATA1*/*Gata1* ^*low/0*^ mice develop fibrosis in the bone marrow.

**FIGURE 8:**
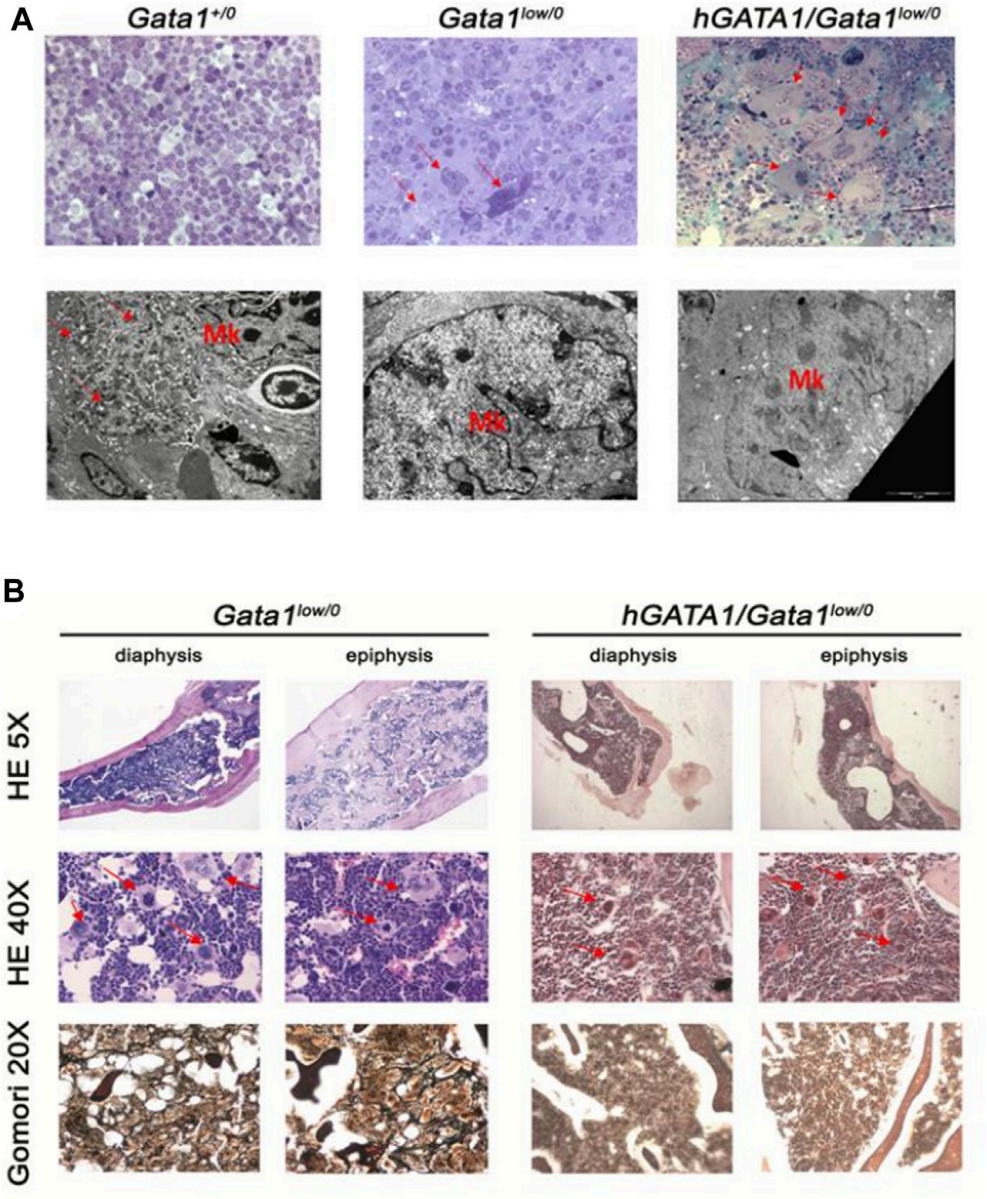
The presence of *hGATA1* rescues fibrosis in the bone marrow of *Gata1* ^*low/0*^ mice without decreasing the great number of abnormally immature megakaryocytes present in their organs. **(A)** Top panels: semithin sections of spleens from representative *Gata1* ^*+/0*^, *Gata1* ^*low/0*^ and *hGATA1/Gata1* ^*low/0*^ mice, as indicated (magnification 40X). By contrast, with *Gata1* ^*+/0*^ mice, megakaryocytes (MK) both from the spleen [arrows in **(A)**] of both *Gata1* ^*low/0*^ and *hGATA1/Gata1* ^*low/0*^ mice are organized in clusters of small cells. Lower panels: transmission electron microscopy analyses of representative megakaryocytes from the spleens of *Gata1* ^*+/0*^, *Gata1* ^*low/0*^ and *hGATA1/Gata1* ^*low/0*^ mice (magnification 4,400X). By contrast with the well organized platelet territories and heavily electron dense granules present in the cytoplasm of wild-type megakaryocytes, the cytoplasm of megakaryocytes from both *Gata1* ^*low/0*^ and *hGATA1/Gata1* ^*low/0*^ mice is poorly partitioned into platelet territories and contains granules with light-electron density, an indication of poor protein content. **(B)** Histological sections of the diaphysis and epiphysis region of femurs from *Gata1* ^*low/0*^ and *hGATA1/Gata1* ^*low/0*^ mice, as indicated. The upper and middle panels present hematoxylin-eosin (HE) staining of representative transversal sections from *Gata1* ^*low/0*^ (left) and *hGATA1/Gata1* ^*low/0*^ (right) mice at 5X (top panels) and 40X (middle panels) magnifications to highlight details of the bone and of the medulla, respectively. The 5X magnification shows that the femur from both *Gata1* ^*low/0*^ and *hGATA1/Gata1* ^*low/0*^ mice contains numerous bone trabeculae in its diaphysis and thicker compact bone sections in its epiphysis, an indication of active osteogenesis while the space inside the bone available for the medulla is limited. The 40X magnification shows the presence in the medulla from both experimental groups of numerous clusters of megakaryocytes (red arrows) similar to those which were observed in the spleen [panel **(A)**]. The Gomori staining in the bottom panels (magnification 20X) indicates presence of fibrosis in the medulla of *Gata1* ^*low/0*^ mice but not in that from *hGATA1/Gata1* ^*low/0*^. littermates. Results are representative of those observed in 3 mice per experimental groups.

In a first set of experiments we confirmed that *hGATA1* does not rescues the megakaryocyte defects induced by the mutation by comparing the frequency of the clusters of megakaryocytes and the ultrastructural morphology of these cells in the spleen and bone marrow from *Gata1* ^*+/0*^, *Gata1* ^*low/0*^ and *hGATA1/Gata1* ^*low/0*^ mice (Figure 8 and data not shown). For reasons linked to technical challenges in performing TEM studies on femurs, ultrastructural studies were performed on spleen only. Previous publications (Vyas et al., 1999; Centurione et al., 2004) had shown that the *Gata1* ^*low*^ mutation blocks megakaryocytic maturation between stage I and II, resulting in accumulation of megakaryocytes arranged in clusters and with a cytoplasm partitioned by a rudimental demarcation membrane system and containing mostly granules of light electron-density instead of the heavy electron-density granules that characterize the cytoplasm of normal megakaryocytes. Electron and light microscopy observations indicated that the *hGATA1* transgene did not rescue the abnormal morphology of the megakaryocytes from the spleen and marrow of *Gata1* ^*low*^ mice. In fact, morphological analysis of spleen from *Gata1* ^*+/0*^, *Gata1* ^*low/0*^ and *hGATA1/Gata1* ^*low/0*^ adult mice showed that, in contrast with wild type mice, in both groups megakaryocytes were organized in clusters of small cells with poorly developed platelet territories partitioned by a rudimental demarcation membrane system and light-electron dense granules (Figure 8A). Histological observations of the femur of these mice groups confirmed the presence of megakaryocytes clusters also in the medulla from both *hGATA1/Gata1* ^*low/0*^ and *Gata1* ^*low/0*^ mice (Figure 8B) while these clusters are absent from the medulla of femurs from wild type animals [(Vannucchi et al., 2002) and data not shown]. The conclusion that the megakaryocytes from *hGATA1/Gata1* ^*low/0*^ and *Gata1* ^*low/0*^ mice are similarly immature is supported also by the observation that the trabecular and the compact bone in both animal groups is thicker than normal (Figure 8B). Increased osteogenesis, in fact, is one of the traits expressed by *Gata1* ^*low*^ mice that has been more strongly mechanistically linked with the megakaryocyte abnormalities induced by the mutation (Kacena et al., 2004).

Collectively, in agreement with the observation that *hGATA1* under the control of *μLCR* coupled with the *β-globin* gene promoter is not expressed in megakaryocytes, the presence of this transgene did not rescue the megakaryocyte abnormalities induced by the *Gata1* ^*low*^ mutation.

To a surprise, however, in spite megakaryocytes of *hGATA1/Gata1* ^*low/0*^ remained immature, the double mutants did not develop fibrosis in bone marrow, providing further indication that the transgene rescues the myelofibrotic trait induced by the *Gata1* ^*low*^ mutation (Figure 8B).

## Discussion

The hypomorphic *Gata1* ^*low*^ mutation impairs expression of the *Gata1* gene already at the levels of hematopoietic progenitor cells and continue to exert its effects in erythroid cells and megakaryocytes (Ling et al., 2018). The consequent reduced content of GATA1 protein results in the following cell specific abnormalities: it increases proliferation of MEP altering their differentiation potential making the cells able to generate mast cells under erythroid-specific conditions (Ghinassi et al., 2007), it increases the apoptotic rates of the erythroblasts (McDevitt et al., 1997; Vannucchi et al., 2001) and blocks megakaryocyte maturation retaining these cells in a proliferative state (Vyas et al., 1999; Vannucchi et al., 2002). As a consequence, the mice are born anemic and thrombocytopenic (McDevitt et al., 1997; Vyas et al., 1999). With age, they recover from their anemia by recruiting the spleen as extramedullary site (Migliaccio et al., 2009; Spangrude et al., 2016) but retain the abnormalities at the level of MEP (Ghinassi et al., 2007) and megakaryocytes (Vyas et al., 1999; Vannucchi et al., 2002) for all their life developing myelofibrosis with age (Vannucchi et al., 2002). In fact, overall, the phenotype of *Gata1* ^*low*^ mice is similar to that of patients with primary myelofibrosis, the most severe of the Philadelphia-negative myeloproliferative neoplasms which is associated with abnormalities in MEP proliferation and megakaryocyte maturation similar to those observed in these mutants (Zahr et al., 2016).

In this manuscript, we used a standard *trans*-complementation gene approach with the *hGATA1* transgene to address two questions: 1) is expression of *hGATA1* erythroid restricted? 2) Since *hGATA1* is not expected to be expressed by megakaryocytes, do the double mutants develop myelofibrosis, a disease driven by abnormalities sustained by reduced *Gata1* expression in these cells?

To address the first question, we investigated whether the *hGATA1* gene under the control of a *μLCR* coupled with the promoter of the *β-globin* gene is expressed in hematopoietic cells from adult mice carrying the *Gata1* ^*low*^ mutation, identified the cells in which the transgene is expressed and analyzed whether the levels of expression sustained by these regulatory sequences are sufficient to rescue the phenotypic abnormalities induced by the mutation. Our results confirmed that embryos carrying the *Gata1* ^*low/0*^ mutation are anemic and thrombocytopenic while adult mutants express significantly lower levels of Hct and platelet counts compared to the wild type littermates. We confirmed that in *Gata1* ^*low/0*^ mice, the levels of Gata1 transcripts are reduced already in progenitor cells (CMP/GMP and MEP) and not only in erythroid and megakaryocyte precursors. We also confirmed that reduced levels of *Gata1* mRNA resulted in lower GATA1 protein content in erythroid cells, which displayed greater apoptotic levels, and in megakaryocytes, that remained morphologically immature. The *hGATA1* under the control of *μLCR*/*β-globin* regulatory sequences was already expressed at the level of progenitor cells (both CMP/GMP and MEP) and of erythroid cells prospectively isolated from *hGATA1/Gata1* ^*low/0*^ littermates. These data are consistent with the observation that the *μLCR*/*β-globin* regulatory sequences are already active at the levels of the stem/progenitor cells, which express low but clearly detectable levels of *β-globin* mRNA, and in erythroid cells but not in cells of other hematopoietic lineages such as megakaryocytes (Beru et al., 1990; Ashihara et al., 1997; McKinney-Freeman et al., 2012). The pattern of cell specificity of the expression of *hGATA1* was correlated with the pattern of the traits induced by the *Gata1* ^*low*^ mutation that were rescued by the presence of the transgene. On one hand, the differentiation potential of MEP prospectively isolated from the double mutants became restricted to cells of erythroid and megakaryocytic lineage having lost their ability to generate mast cells. In addition, erythroid cells expressed nearly normal levels of the erythroid specific genes analyzed and displayed low apoptotic rates. On the other hand, megakaryocytes remain detected in great numbers, an indication of increased proliferation, express altered levels of the megakaryocyte specific genes analyzed and displayed an immature morphology. Consistently with the rescue of the MEP alterations, erythropoiesis was effective in the bone marrow of the double mutant mice making their extramedullary hematopoiesis in spleen dispensable for survival. Consistently with the rescue of the defective erythroid maturation, the animals were not anemic and, as predicted by the failure of the transgene to rescue the megakaryocyte defects, the mice remained thrombocytopenic and with increased osteogenesis, a trait driven by the abnormal megakaryocytes (Kacena et al., 2004).

The observation that the expression of *hGATA1* driven by the *μLCR*/*β-globin* regulatory sequences is restricted to the erythroid cells has important implications for gene therapy. In fact, suitable gene therapy approaches require robust evidence not only for the safety of the retroviral vector used, but also for the specificity of its expression, essential to minimize its off-target effects (Demirci et al., 2018; High and Roncarolo, 2019). Based on the strong evidence for erythroid specificity displayed by the *μLCR*/*β-globin regulatory* sequences in transgenic mouse models (Li and Stamatoyannopoulos, 1994; Papayannopoulou et al., 2000; Li et al., 2002), these sequences are used to drive the expression of the human *β*-globin in most of the retroviral vectors currently under investigation of gene therapy of hemoglobinopathies (Wang et al., 2019; Morgan et al., 2020). Our results confirm that these regulatory sequences are indeed erythroid specific (and therefore likely to be safe) because, with the exception of some expression of the transgene at the level of progenitor cells, in which even under normal circumstances these regulatory sequences are active (McKinney-Freeman et al., 2012), they drove expression of the transgene mainly in erythroid cells and were ineffective in the closely related megakaryocytes.

Erythroid commitment and differentiation is sequentially driven by two genes of the GATA transcription factors family, *GATA1* and *GATA2*. *GATA2* is expressed at high levels in multipotential progenitors, affecting their expansion and guiding the early stage of erythroid commitment (Tsai et al., 1994). As erythroid commitment progresses, *GATA2* activates the expression of *GATA1* which is abundantly expressed in late progenitor cells and erythroblasts (Leonard et al., 1993). The switch from *GATA2* to *GATA1* expression in the control of erythropoiesis is known as the GATA switch and represents the clock which initiates the translation of the erythroid specific genes (Katsumura and Bresnick, 2017; Bresnick et al., 2020). Once activated by GATA2, the expression of *Gata1* is self-sustained. In mice, expression of *Gata1* is regulated by three DNase hypersensitive sites (HS), two of which (HSI and HSII) lay within 8 Kb upstream of the coding sequences and the third one (HSIII) within the first intron (Kobayashi and Yamamoto, 2007). The *Gata1* ^*low*^ mutation specifically deletes HSI. The fact that erythroid cells from *Gata1* ^*low*^ mice express some level of the endogenous gene reflects the positive effects on its translation exerted by GATA2 likely by binding to HSII and/or HSIII. The observation that in the double mutant cells increases in the content of the GATA1 protein due to the contribution of *hGATA1* does not increase the expression of the endogenous gene confirms that HSI is an indispensable element of the self-sustained regulatory loop of *Gata1* transcription the expression of which remains at the levels driven by GATA2.

To address the second question, we investigated whether *hGATA1/Gata1* ^*low/0*^ mice develop myelofibrosis with age. To a surprise, in spite the megakaryocytes of the double mutants remained GATA1 hypomorphic, these mice did not develop hematopoietic failure and fibrosis in their bone marrow, i.e., the two distinctive traits for myelofibrosis. This observation is counterintuitive given the strong evidence that both in patients and in causative mutation-driven animal models, myelofibrosis is sustained by abnormal GATA1 hypomorphic megakaryocytes (Vannucchi et al., 2005; Gilles et al., 2017). In addition, megakaryocyte-restricted expression of the driver mutations is necessary and sufficient to induce myelofibrosis even if the hematopoietic stem cells are normal (Jeremy Wen et al., 2015; Zhan et al., 2016). This apparent contradiction has been recently clarified by new exciting single cell profiling data indicating that the megakaryocytes in the bone marrow are a mixture of three populations, each one exerting a different function (Psaila et al., 2020; Yeung et al., 2020; Migliaccio and Hoffman, 2021; Sun et al., 2021; Wang et al., 2021). In fact, in addition to megakaryocytes poised to form platelets, the hematopoietic stem cells also generate megakaryocytes poised to exert immune-functions in the lungs (Pariser et al., 2021) or niche-functions locally, by secreting collagen and other extracellular matrix proteins (Malara et al., 2014). This single cell profiling also indicated that while maturation of platelet-poised and immune-poised megakaryocytes requires upregulation of *GATA1* expression, niche-poised megakaryocytes are dependent on low levels of GATA1 (Wang et al., 2021). Data from the Balduini laboratory indicate that megakaryocytes expressing collagens, which are supposedly the niche-poised cells, are very rare in the bone marrow from healthy individuals at opposite with myelofibrosis patients where a great proportion of megakaryocytes in the bone marrow express collagen (Abbonante et al., 2016), suggesting that niche poised-megakaryocytes may contribute to deposition of fibrosis in the bone marrow. Overall, these recent results suggest the hypothesis that hypomorphic GATA1 content at the levels of megakaryocytes precursors switches their fate from platelet-poised to niche-poised cells, potentially contributing to fibrosis. Our data are consistent with this refinement of the pathobiological role of megakaryocyte abnormalities in myelofibrosis. In fact, the rescue of the myelofibrotic phenotype of the *Gata1* ^*low*^ mice is associated with normalization of the abnormal differentiation potential of the stem/progenitor cells (which express *hGATA1* and became able to differentiate in the bone marrow) and although expression of *hGATA1* is below detection in megakaryocytes prospectively isolated from the bone marrow, it is expressed by megakaryocytes differentiated in culture from the double mutants, the large majority of which has been suggested to represent either niche- or immune-poised MKs (Malara et al., 2014). It is then possible that expression of *hGATA1* at the level of the multi-potent megakaryocyte precursors prevented their switch to niche-poised cells, thereby rescuing the myelofibrosis trait. Unfortunately, different populations of megakaryocyte precursors poised to generate megakaryocytes and immune-poised or niche-poised megakaryocytes have all the same morphology (Wang et al., 2021; Migliaccio and Hoffman, 2021), thus they cannot be discriminated by morphological observations. This novel pathobiological model may only be tested when cell surface markers to prospectively identify the various cell populations will became available.

Last but not least, our data have implications to design strategies for the cure of primary myelofibrosis, the most severe of the Philadelphia-negative myeloproliferative neoplasms which has presently no cure (Zahr et al., 2016). In fact, drugs targeting the driver mutations displayed by the malignant hematopoietic stem clones, such as the JAK1/2 inhibitor ruxolitinib, are greatly effective in ameliorating the clinical manifestation of the disease but it is still unclear whether they are also effective in halting the progression toward its final stage (Mascarenhas and Hoffman, 2013; Tefferi, 2021). As of today, with exception of bone marrow transplantation which may be offered to a limited number of patients (Tamari et al., 2015), this disease is still an unmet clinical need. Based on the observation that bone marrow transplantation cures the disease by rescuing both the hematopoietic stem cell and the microenvironmental abnormalities displayed by the patient, the consensus has been reached that treatment of the disease requires the use of drug combinations targeting both abnormalities (Eran et al., 2019; Tefferi, 2021). Our data support this consensus and indicate that these combination therapies should not combine drugs targeting the driver mutations (such as ruxolitininb) with those that target the platelet-poised megakaryocytes but rather with those that target the niche-poised cells.

## Data Availability

The original contributions presented in the study are included in the article/Supplementary Materials, further inquiries can be directed to the corresponding author.
