# Supplementary material for: hGATA1 Under the Control of a μLCR/β-Globin Promoter Rescues the Erythroid but Not the Megakaryocytic Phenotype Induced by the Gata1 low Mutation in Mice
Source: Front Genet. 2021 Oct 11;12:720552. doi: 10.3389/fgene.2021.720552 (PMC8542976; doi:10.3389/fgene.2021.720552)
Supplement: Supplementary file 1 [file DataSheet1.docx]

Supplementary Materials

Supplementary Figure
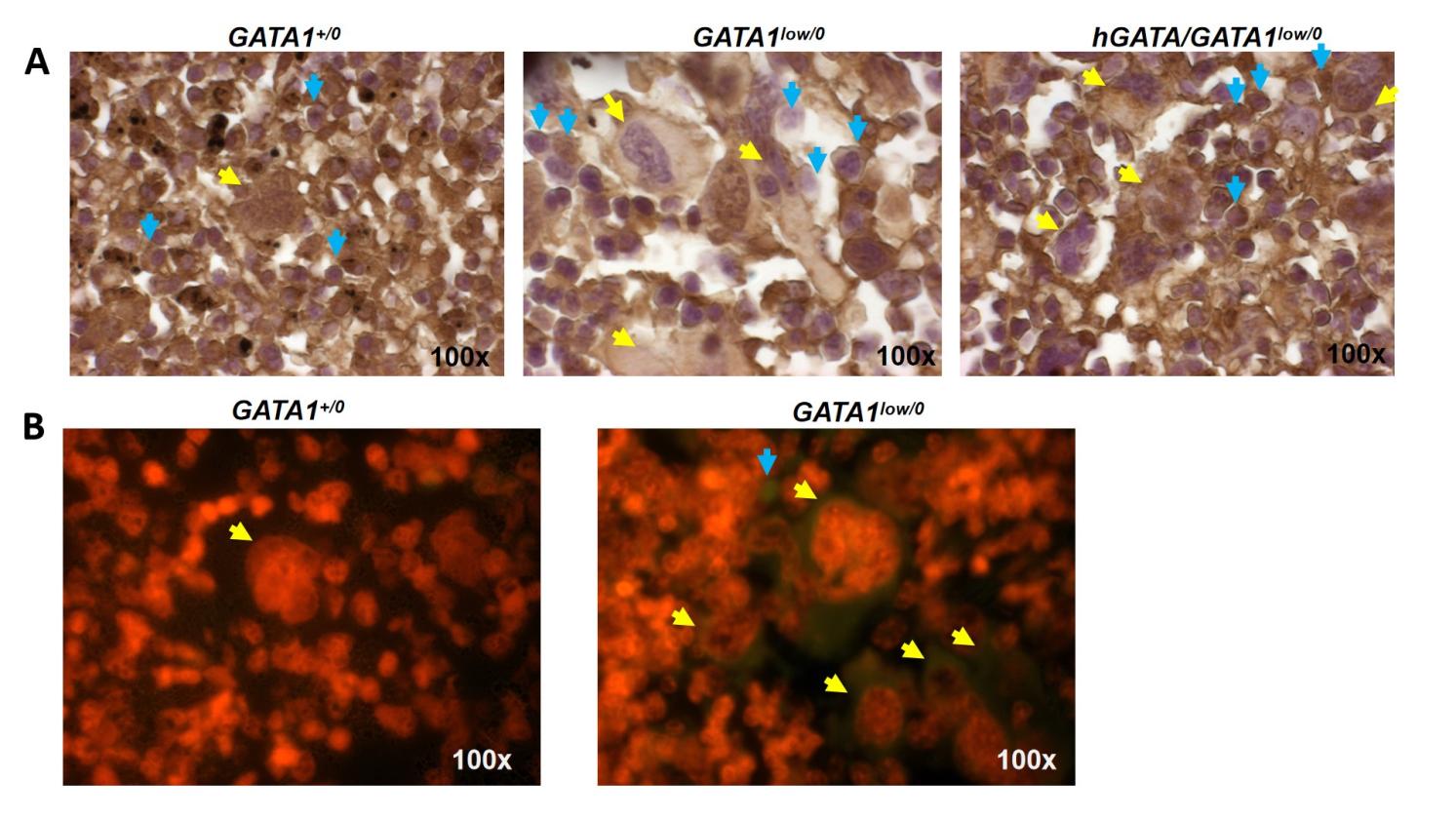


**Figure S1 A-B**

**Figure S1A:** **Erys (blue arrows) but not MKs (yellow arrows) from adult *hGATA1/Gata1^low/0^* contain detectable levels of GATA1 protein.** Immunohistochemical staining with a GATA1 antibody of sections from the spleen of *Gata1^+/0^, Gata1^low/0^* and *hGATA1/Gata1^low/0^* mice. In the Erys of *Gata1^+/0^* and *hGATA1/Gata1^low/0^* mice, the GATA1 staining is detectable in the nucleus. In the MKs from *Gata1^+/0^* mice, it is detectable both in the nucleus and cytoplasm. The figures are representative of those obtained with 2 additional mice per experimental group. Magnification 100X in all the panels.

**Figure S1B:** TUNEL staining of sections from the spleen of *Gata1^+/0^* and *Gata1^low/0^* mice. Blue and yellow arrows indicate Erys and MKs, respectively.To be noted the peri-nuclear localization of the TUNEL staining in the MKs. The fact that Erys are small and contain large nuclei prevents a clear localization of the TUNEL staining. Magnification 100X in all the panels.
